# AP39, a Mitochondria-Targeted Hydrogen Sulfide Donor, Supports Cellular Bioenergetics and Protects against Alzheimer's Disease by Preserving Mitochondrial Function in APP/PS1 Mice and Neurons

**DOI:** 10.1155/2016/8360738

**Published:** 2016-01-31

**Authors:** Feng-li Zhao, Fang Fang, Pei-feng Qiao, Ning Yan, Dan Gao, Yong Yan

**Affiliations:** ^1^Experimental Research Center, Department of Neurology, The First Affiliated Hospital of Chongqing Medical University, Chongqing Medical University, Chongqing 400016, China; ^2^Department of Neurology, The University-Town Hospital of Chongqing Medical University, Chongqing 400016, China

## Abstract

Increasing evidence suggests that mitochondrial functions are altered in AD and play an important role in AD pathogenesis. It has been established that H_2_S homeostasis is balanced in AD. The emerging mitochondrial roles of H_2_S include antioxidation, antiapoptosis, and the modulation of cellular bioenergetics. Here, using primary neurons from the well-characterized APP/PS1 transgenic mouse model, we studied the effects of AP39 (a newly synthesized mitochondrially targeted H_2_S donor) on mitochondrial function. AP39 increased intracellular H_2_S levels, mainly in mitochondrial regions. AP39 exerted dose-dependent effects on mitochondrial activity in APP/PS1 neurons, including increased cellular bioenergy metabolism and cell viability at low concentrations (25–100 nM) and decreased energy production and cell viability at a high concentration (250 nM). Furthermore, AP39 (100 nM) increased ATP levels, protected mitochondrial DNA, and decreased ROS generation. AP39 regulated mitochondrial dynamics, shifting from fission toward fusion. After 6 weeks, AP39 administration to APP/PS1 mice significantly ameliorated their spatial memory deficits in the Morris water maze and NORT and reduced A*β* deposition in their brains. Additionally, AP39 inhibited brain atrophy in APP/PS1 mice. Based on these results, AP39 was proposed as a promising drug candidate for AD treatment, and its anti-AD mechanism may involve protection against mitochondrial damage.

## 1. Introduction

Alzheimer's disease (AD) is the most universal age-related neurodegenerative disease and form of dementia, affecting approximately 5.3 million American people. Of those, 5.1 million are over the age of 65 years [1]. AD initially causes memory impairment, and as the disease progresses, patients exhibit motor aberrancies, personality changes, language deficiencies, and other neuropsychiatric symptoms. Because AD is a complex and multifaceted disease involving various mechanisms, including energy metabolism [2], inflammation, and abnormal cell cycle control [3], the currently available treatments include a combination treatment regimen targeting two or more aspects of AD pathology [4]. For example, drug treatment for AD is primarily based on the acetylcholine hypothesis or the amyloid-*β* (A*β*) accumulation hypothesis, but none of these drugs can stop or reverse the progression of AD.

Swerdlow first proposed the “AD mitochondrial cascade hypothesis” in 2004 [5]. Recently, increasing evidence has shown that mitochondrial dysfunction is a prominent factor in AD pathogenesis [6], as either a cause or a consequence of A*β* toxicity. Severe structural and functional abnormalities of the mitochondria were observed in the immediate vicinity of A*β* plaques [7]. Moreover, A*β* gradually accumulates within the mitochondria of both living mouse models of AD and human AD brain sections, and A*β* has been found to directly interact with several mitochondrial proteins, for example, cyclophilin D (CypD), amyloid-*β* binding alcohol (ABAD) dehydrogenase, and dynamin-related protein 1 (Drp1) [8]. These interactions impair the physiological functions of mitochondria, resulting in limited electron transfer, abnormal adenosine 5′-triphosphate (ATP) production, increased production of reactive oxygen species (ROS), and altered mitochondrial morphology and mobility in AD transgenic models [9, 10]. Furthermore, a growing body of research suggests that mitochondrial biogenesis is considerably decreased in the brains of both AD patients and AD model mice [9, 11–13]. Impaired mitochondrial biogenesis is conducive to mitochondrial dysfunction in AD [13].

Hydrogen sulfide (H_2_S) has recently been recognized as an endogenous gaseous mediator that plays multiple regulatory roles in humans and mammals [14]. Increasing evidence has demonstrated that the dysregulation of H_2_S homeostasis is implicated in the pathological processes of AD. Recently, our experimental research has revealed that the level of H_2_S is decreased in AD patients and that this change in the H_2_S levels may be related to the severity of AD. The emerging mitochondrial roles of H_2_S include antioxidant, antiapoptotic, and anti-inflammatory effects [15]. Tang revealed that H_2_S modulates A*β*-induced damage to PC12 cells by ameliorating the decrease in the mitochondrial membrane potential** (**MMP, ΔΨm**)** and attenuating the increase in intracellular ROS levels [16]. Moreover, Xuan et al. found that H_2_S may attenuate spatial memory impairment and neuroinflammation within the hippocampus of AD model mice [17]. With regard to the regulatory role of H_2_S in cellular bioenergy metabolism, recent studies have shown that H_2_S can serve as a physiological electron donor and as an inorganic energy source in mammalian cells. Recently, Módis et al. reported that an endogenous intramitochondrial H_2_S-producing pathway, which is governed by 3-mercaptopyruvate sulfurtransferase (3-MST), complements and balances the bioenergetic activity of Krebs cycle-derived electron donors in the Hepa1c1c7 cell line [18].

Taken together, these findings indicate that the potential effects of H_2_S on mitochondrial dysfunction in AD models are significant. Therefore, to clarify whether the new H_2_S donor AP39, which contains the mitochondria-targeting compound triphenylphosphonium (TPP+) coupled to an H_2_S-donating moiety (dithiolethione) via an aliphatic linker, regulates mitochondrial function and cellular bioenergetics is of great interest. Using APP/PS1 neurons and mice, we first detected the overall viability of the neurons and evaluated the effects of AP39 on cellular bioenergetics and mitochondrial function. In addition, we examined the effects of AP39 on spatial memory deficits and on magnetic resonance imaging (MRI) characteristics of the mouse brains.

## 2. Materials and Methods

### 2.1. Materials

AP39, a novel mitochondria-targeted H_2_S donor, was designed and synthesized by Medicilon Inc., as described previously [19, 20]. Antimycin A, 0.25% trypsin, 2-deoxyglucose, oligomycin, carbonyl cyanide-4-(trifluoromethoxy)phenylhydrazone (FCCP), trichloroacetic acid, rotenone, 7-azido-4-methylcoumarin (AzMC), monobasic potassium phosphate, dibasic sodium succinate hexahydrate, adenosine 5′-triphosphate (ATP) disodium salt hydrate, fatty acid-free BSA, sodium hydrosulfide hydrate (NaSH·*x*H_2_O), N,N-diethyl-p-phenylenediamine sulfate, magnesium chloride, and iron(III) chloride (FeCl_3_) were purchased from Sigma-Aldrich Company. The monoclonal anti-A*β* antibody 6E10 was obtained from Covance Inc. (Princeton, NJ, USA). Drp1 was purchased from Novus Biologicals, Inc. Fis1 was purchased from Proteintech Group. Mfn1 and Mfn2 antibodies were purchased from Santa Cruz Biotech, and OPA-1 antibody was purchased from BD Transduction Laboratories. The secondary antibodies goat anti-mouse-HRP and donkey anti-rabbit-HRP antibodies were purchased from GE Healthcare. The Pierce BCA protein assay kit was purchased from Thermo Fisher Scientific (Waltham, MA, USA). Dulbecco's modified Eagle's medium (DMEM), 1% glutamine, and 1% penicillin were obtained from Invitrogen. All other chemicals were purchased from Amresco (Solon, OH, USA).

### 2.2. Animals and Treatments

This experiment was performed as described previously [21]. All animal protocols were approved by the Animal Research Committee of Chong Qing Medical University. Heterozygous APP/PS1 double-transgenic mice (APPswe-PS1dE9) were crossed with nontransgenic female mice (the Model Animal Research Center of Nanjing University, Jiangsu, China) to produce hemizygous transgenic mice and nontransgenic littermates. In the double-transgenic mice that coexpressed the Swedish mutation of APP (APPswe) and two FAD-PS1 variants, A*β* plaques accumulated in the brain, and learning and memory ability declined over time [22]. The mice were housed at 23–25°C with 60% humidity under 12 : 12 h light-dark cycles and were provided with free access to food and water throughout the experiment. Pregnant females were sacrificed at gestational days 15-16 (E15-16), and the embryos were removed for the preparation of brain neuronal cultures. The genotyping for APP and PS1 was performed according to the literature to assign cultures to transgenic and nontransgenic groups according to the PCR results with genomic DNA. For the Morris water maze test and the novel object recognition task (NORT), these 12-month-old mice were divided into four groups of fifteen mice in each group: wild-type (WT) mice treated with deionized water or 100 nM/kg AP39 and AD model mice treated with deionized water or 100 nM/kg AP39. The mice were treated once daily via intraperitoneal injection for 6 weeks prior to the experiments. After the behavioral tests, the mice were scanned by MRI and then sacrificed for the collection of their blood and brains. The blood was collected into heparinized tubes and then centrifuged at 1000 g for 10 min at 4°C. The plasma (supernatant) was collected to detect A*β*_40_ and A*β*_42_ by ELISA. The brains were removed and collected to examine A*β* deposition by immunohistochemistry.

### 2.3. Primary Neuron Culture

Primary cultures of cortical neurons were generated from the brains of individual embryos at E15-16. Briefly, the brains were removed and then placed in Hank's balanced salt solution at 4°C. The cortex was dissected from the brain, chopped, and digested in 0.25% trypsin for 16 min at 37°C with gentle shaking. For the mixed neuronal cultures, after cell counting, the dissociated cells were plated at a density of 2 × 10^5^ cells/cm^2^ in a 35 mm dish on poly-d-lysine-coated cover slips in DMEM containing 10% F-12 and 10% fetal bovine serum (FBS) and were maintained at 37°C in a humidified atmosphere of 95% air and 5% CO_2_. After culturing for 24 h in vitro, the medium was replaced with serum-free neurobasal medium containing 2% B27 serum-free supplement, 1% glutamine, and 1% penicillin. Thereafter, half of the medium was replaced with neurobasal medium every three days. Pure neuronal cultures were supplemented with glia-conditioned medium (GCM). These neurons were prepared in a similar manner, except that cytosine arabinoside (5 *μ*M) was added to the culture media at DIV 4 to block the proliferation of glia, and the cultures were maintained in GCM. After 16 days, the neurons were treated with different concentrations of AP39 diluted in the appropriate medium for 24 h.

### 2.4. Measurement of H_2_S Production

#### 2.4.1. H_2_S Production in Neurons and Brain Tissue (Methylene Blue Assay)

This experiment was performed based on previous literature [23, 24]. Neurons were scraped, collected, and then washed 3 times with phosphate-buffered saline (PBS). The neurons were suspended in ice-cold 50 mM Tris-HCl buffer and homogenized by sonication. Homogenized samples were centrifuged at 10,000 g for 10 min at 4°C, and the supernatants were collected as cell lysates. The protein concentrations of the supernatants were determined using the Quick Start Protein Assay Kit.

We used spectrophotometric measurements based on the formation of methylene blue by H_2_S to detect H_2_S production. We performed all reactions in duplicate. Cell lysates (310 *μ*L, 1 mg/mL) in 1.5-mL tubes were mixed with trichloroacetic acid (20% w/v, 60 *μ*L), zinc acetate (2% w/v, 30 *μ*L), and N, N-dimethyl-p-phenylenediamine sulfate (NNDPD) (20 mM; 40 *μ*L) in 7.2 M HCl and FeCl_3_ (30 mM; 30 *μ*L) in 1.2 M HCl. The optical absorbance of the resulting solution (670 nm) was measured after 15 min using a 96-well microplate reader (Tecan Systems Inc., Switzerland). H_2_S was calculated against a calibration curve of NaHS.

To explore the effect of AP39 on the generation of H_2_S in mice, these 12-month-old WT or APP/PS1 mice were treated with different concentrations of AP39 (25 nM/kg–250 nM/kg). The mice were treated once daily via intraperitoneal injection for 6 weeks prior to determining H_2_S concentration. In brief, mice from each group were anesthetized with chloral hydrate and euthanized by decapitation. Tissues from the hippocampus and cerebral cortex were immediately removed and then homogenized in ice-cold 50 mM potassium phosphate buffer (12% wt./vol, pH 8.0) with a Polytron homogenizer. The homogenates were centrifuged at 47,000 ×g for 10 min at 4°C and supernatants were collected. Then the H_2_S production was measured as above described via methylene blue assay.

#### 2.4.2. H_2_S Detection in Mitochondria

Neurons were seeded at a density of 5 × 10^4^ cells/well in a Lab-Tek II chamber coverglass system and were maintained at 37°C in a humidified atmosphere of 95% air and 5% CO_2_. The H_2_S-sensitive fluorescent dye AzMC was incorporated into a cell-based assay to detect H_2_S production [19, 25]. The neurons were loaded with the fluorogenic dyes 10 *μ*M AzMC and 200 nM MitoTracker Red CMXRos (Invitrogen) at 37°C for 30 min. Different concentrations of AP39 were added to fresh media, and the neurons were further incubated for 2 h, after which H_2_S imaging was performed. After washing 3 times with PBS, the specific fluorescence from the various dyes was examined using a confocal laser scanning microscope.

### 2.5. Bioenergetic Analysis in Neurons

We used the XF24 Extracellular Flux Analyzer (Seahorse Bioscience, Billerica, MA, USA) to measure cellular bioenergetic function as previously described [26]. Neurons were treated with different concentrations of AP39 in medium. Neurons were seeded at the optimal density of 3.5 × 10^4^ cells per well and were maintained at 37°C in a humidified atmosphere of 95% air and 5% CO_2_. Next, the indices of mitochondrial function were measured according to the following protocol. After recording the basal OCR and PPR levels, oligomycin (1.5 *μ*g/mL) was used to assess the mitochondrial ATP production rate, and FCCP (0.5 *μ*M) was used to assess the maximal mitochondrial respiratory capacity (a parameter that characterizes overall mitochondrial function in neurons) via the measurement of the oxygen consumption rate (OCR). Finally, antimycin A (2 *μ*g/mL) and rotenone (2 *μ*M) were used to inhibit the flux of electrons through complexes III and I and to detect the residual nonmitochondrial OCR, which is considered to be mediated by cytosolic oxidase enzymes. Bioenergetic parameters were normalized to the neuron count, as the same number of neurons was seeded in each well.

### 2.6. MTT Assay

Cell viability was determined by the MTT reduction assay method as follows. The neurons were cultured and treated with different concentration of AP39 for 24 h in 96-well plates. MTT was added to each well with a final concentration of 0.5 mg/mL for 4 h. After a 4-h incubation at 37°C, the MTT solution was removed and the insoluble formazan crystal was dissolved in DMSO. The absorbance of the colored solution was measured at 570 nm using a microplate reader (Tecan, USA).

### 2.7. LDH Assay

Lactate dehydrogenase (LDH) release, an indirect measurement of cell death, was determined using a cytotoxicity assay according to the manufacturer's instructions. Briefly, 30 *μ*L of supernatant was saved before the addition of MTT and mixed with 100 *μ*L of freshly prepared LDH assay reagent. LDH release into the culture medium was detected using a colorimetric reaction reading of absorbance at 490 nm according to manufacturer's protocol for the LDH assay kit (Beyotime, Jiangsu, China). LDH activity values are shown as *V*_max_ for kinetic assays in mOD/min.

### 2.8. Cellular ATP Measurements

Neurons were plated at a density of 0.5 × 10^4^ cells/well in 96-well plates before the experiment. WT and APP/PS1 neurons were treated with water or 100 nMAP39, and the samples were measured in triplicate. After incubation for 24 h, the ATP levels were assessed using the ATP Bioluminescent Assay Kit (Sigma-Aldrich). The fluorescence intensity, which was linearly related to the ATP concentration, was measured using a microplate luminometer [27].

### 2.9. Measurement of Mitochondrial and Nuclear DNA Integrity

DNA integrity was assessed using gene-specific semiquantitative PCR assays as described previously [28]. Briefly, total DNA from experimental neurons was isolated using the DNase Blood and Tissue Kit. Quantification of a PCR-amplified 9-kb nuclear-specific DNA fragment using PicoGreen fluorescent dye to measure double-stranded DNA was used to estimate damage to nuclear DNA. Quantification of a PCR-amplified 10-kb mitochondrial-specific DNA fragment using PicoGreen dye was used to detect damage to mitochondrial DNA. The obtained data were normalized by the PCR amplification of a 117-bp mitochondrial genome-specific fragment to correct for multiple copies of the mitochondrial genome. Preliminary assays were performed to ensure the linearity of PCR amplification with respect to the number of cycles and DNA concentration.

### 2.10. Measurement of Intracellular ROS

The fluorescent dye 2′, 7′-dichlorofluorescein diacetate (DCFH-DA) was used to assess the intracellular production of ROS according to the manufacturer's instructions. Following the indicated treatment, the neurons were harvested, washed twice with PBS, and centrifuged at 1000 g for 10 min. Then, the cells were resuspended in 10 *μ*M DCFH-DA (Beyotime, Jiangsu, China) solution and incubated in a CO_2_ incubator at 37°C for 30 min in the dark, followed by three washes with PBS. Finally, the cells were resuspended in 0.5 mL of PBS, and the fluorescence intensity of the samples was analyzed by flow cytometry.

### 2.11. Western Blot

Approximately 1 × 10^7^ neurons were collected for each experiment. The cell samples were lysed with Western and IP lysis buffer (Beyotime, Jiangsu, China) and centrifuged at 12,000 g for 15 min. The protein concentrations were determined using the BCA protein assay kit. A total of 20 *μ*g protein was loaded per lane and subjected to electrophoretic separation in a 10% SDS-PAGE gel. After separation, the proteins were electrically transferred to a nitrocellulose transfer membrane (PerkinElmer Life Sciences) for 1 h. After blocking with 5% nonfat milk in Tris-buffered saline containing Tween-20 (TBST), the membranes were incubated in primary antibodies against Mitofusin-1 (Mfn-1, 1 : 200, rabbit polyclonal), Mitofusin-1 (Mfn-2, 1 : 200, rabbit polyclonal), Optic Atrophy-1 (OPA-1, 1 : 400, mouse monoclonal), Fission-1 (Fis-1, 1 : 1500, rabbit polyclonal), Drp-1 (1 : 200, rabbit polyclonal), or VDAC (1 : 500 to 1 : 1000) overnight at 4°C. The membranes were subsequently incubated in donkey anti-rabbit or goat anti-mouse antibodies for 1 h at 37°C. Chemiluminescent detection was performed using the Pierce ECL Western blotting substrate, and the signals were analyzed using Quantity One software.

### 2.12. Behavioral Testing

#### 2.12.1. Morris Water Maze Test

The Morris water maze test was performed according to the protocols of a previous study [29]. Animals were randomly assigned to different treatment groups and then were pretreated with once-daily administration of either water or AP39 for 6 weeks prior to testing. Briefly, the mice were put into each quadrant in a random order to locate a hidden platform within 1 min, and the experimenter would guide them to the platform if the mice failed to find the platform. Then, the mice were left on the platform for 30 s to help them remember the platform location. The mice were trained to locate the platform using a two-trial-per-day regimen, and the training was divided into two blocks of four trials. The first block of four trials was performed in the morning, and the second block of four trials was performed in the afternoon. In the probe trial, the platform was removed, and the mice were placed in the water for a single 60-s period. The latency to reach the platform was recorded for both the training and probe trials, and the parameters were analyzed using an EthoVision 3.1 analysis system by an observer who was blinded to the treatment status of the mice.

#### 2.12.2. Novel Object Recognition Task (NORT)

The novel object recognition task (NORT), which is based on the natural instinct of rodents to interact more with a novel object than a familiar object, was performed to assess the memory of the experimental mice as described in the literature [30]. Briefly, on the first day of acclimation, each mouse was provided with 10 min to interact with either object for combined 30 s. The total distance traveled was recorded and analyzed to evaluate the motor ability of the mice. On the following day, the mice were exposed to two identical objects (A1 and A2) for 10 min for familiarization. Both objects A1 and A2 had identical textures, colors, and sizes and were positioned in two adjacent corners from the walls. After a 24-h delay in the mice cages, the mice were again placed into the same arena used before for 10 min and then exposed to the field for 5 min in the presence of the old familiar (A1) and a new different novel (B) object. We determined the amount of time spent at both familiar and novel objects through video analysis using a “within object area” scoring method. A mouse was scored as interacting with the object when its nose was in contact with the object or directed at the object within ≤ 2 cm. Time spent standing, sitting, or leaning on the secured object was not scored. The exploratory preference was defined as the percentage of total time that the animal spent investigating the novel object and calculated for each animal by the following ratio: TB/(TA + TB) × 100% [TA: time spent exploring the familiar object A; TB: time spent exploring the novel object B].

### 2.13. Mouse Brain MRI

The mice were scanned by MRI after treatment with water or AP39 for 6 weeks as described previously in the literature [31]. The MRI experiment was conducted in Da Ping Hospital, Research Institute of Surgery, Third Military Medical University, China. The mice were anesthetized with 10% chloral hydrate-saline and then placed in the prone position. Their chests were connected to a life signal detector to monitor their physiological status. The heads of the mice were scanned using a 7.0 T ultrahigh field animal MRI scanner (7.0 T ClinScan system, Bruker BioSpin, Ettlingen, Germany). T2-weighted images of the mouse heads were acquired from the sagittal plane, axial direction, and coronal plane according to the following conditions: TR = 3200 ms, TE = 45 ms, field of view (FOV) = 22 × 22 mm, slice thickness = 0.5 mm, a scan matrix = 384 × 384, and repetitions (NEX) = 5.

### 2.14. ELISA for A*β*_40_ and A*β*_42_

A commercially available ELISA kit for A*β*_40_ and A*β*_42_ was purchased from Invitrogen. The plasma from samples was collected by centrifugation (1,000 g at 4°C for 10 min), and 100 *μ*L of plasma was used for A*β*_40_ and A*β*_42_ measurement. The secreted levels of A*β*_40_ and A*β*_42_ in blood were quantitatively measured according to the manufacturer's instructions.

### 2.15. Immunohistochemistry

After the behavioral tests, the mice were deeply anaesthetized with chloral hydrate (400 mg/kg body weight) and sacrificed for the preparation of brain slices [32]. The brains were removed from the skull, postfixed overnight in 4% paraformaldehyde at 4°C, and transferred to 30% sucrose solution for dehydration. Subsequently, the brains were frozen and sliced into 30-mm thick sections using a Leica CM3050 S cryostat. After multiple washes in PBS, the slices were incubated with a 6E10 primary antibody (1 : 1,000 diluted) in 10% goat serum overnight at 4°C. The tissue sections were washed 3 times for 10 min in PBS and then incubated with a TRITC-conjugated goat anti-mouse IgG antibody at room temperature for 1 h followed by PBS washing. The slides were subsequently stained with diaminobenzidine and counterstained with hematoxylin. The images were acquired using a 10x objective on a Carl Zeiss LSM780 confocal microscope (Olympus BX60, Tokyo, Japan). The A*β* plaque areas were quantified using Image-Pro Plus 6.0 software.

### 2.16. Statistical Analysis

All statistical analyses were performed using SPSS 22.0 software. All data were presented as the means ± SEM. The data in the Morris water maze were analyzed using two-way ANOVA coupled with a post hoc Fisher's LSD test using GraphPad Prism 6.0 software. The data from other experiments were analyzed by one-way ANOVA coupled with Dunnett's posttest using GraphPad Prism 6.0 software, and *P* ≤ 0.05 was considered to be statistically significant.

## 3. Results

### 3.1. AP39 Increased the Generation of H_2_S in Neurons and Mitochondria

**Figure 1:**
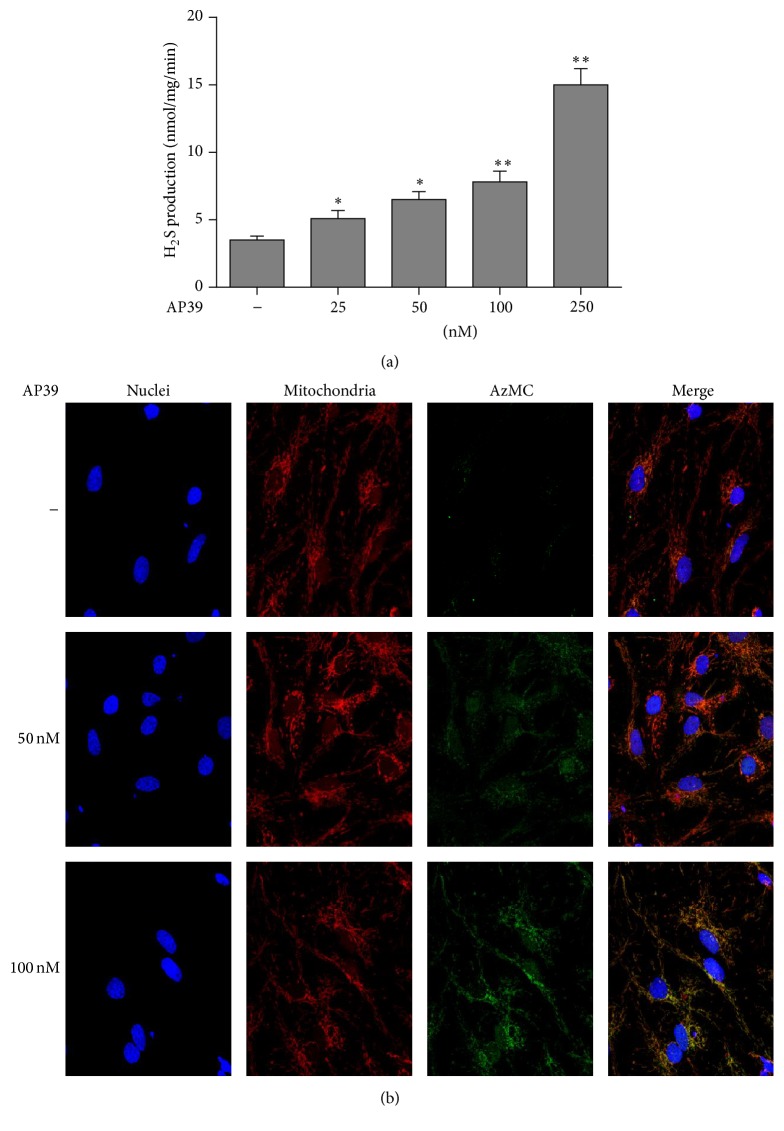
AP39 generates H_2_S in WT neurons, primarily in the mitochondria. (a) The contribution of AP39 to H_2_S production in neurons. Neurons from WT mice were treated with various concentrations of AP39 for 2 h, and H_2_S production was detected by methylene blue assay. (b) Neurons were treated with different concentrations of AP39 for 2 h, and intracellular H_2_S was detected using the fluorescent probe AzMC. DAPI was used to stain nuclei, and MitoTracker was used to stain mitochondria. The colocalization of H_2_S with mitochondria was indicated by the overlapping of red (mitochondria) and green (H_2_S) fluorescence in the merged image. Note the concentration-dependent increase in the H_2_S signal in response to AP39 treatment. ^*∗*^ *P* < 0.05, ^*∗∗*^ *P* < 0.01 compared with control treatment (no AP39).

H_2_S levels were comparable between WT neurons treated with different concentrations of AP39 for 2 h. AP39 (25–250 nM) induced a concentration-dependent increase in H_2_S generation and in the fluorescence of the H_2_S-detecting dye AzMC, and the signal was significantly colocalized with the mitochondria (Figures 1(a) and 1(b)). Notably, baseline fluorescent signal was detectable in the neurons. In this study, the low basal levels of H_2_S did not indicate a mitochondrial preference (Figure 1(b)). In conclusion, the data in Figure 1 indicate that AP39 contributes to the synthesis of H_2_S in neurons, especially in the mitochondria.

### 3.2. Biphasic Effects of AP39 on Cellular Bioenergetics

**Figure 2:**
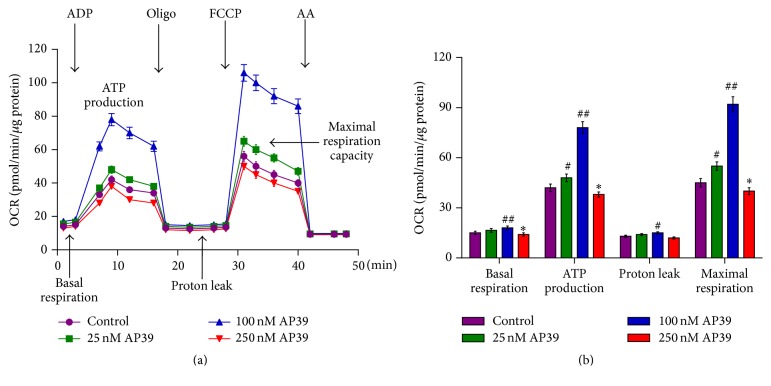
Biphasic effects of AP39 on the cellular bioenergetics of the WT neurons. Neurons from WT mice were incubated in AP39 (25-250 nM) for 2 h, and bioenergetic parameters were measured using an Extracellular Flux Analyzer. (a) Representative tracings. (b) The calculated bioenergetic parameters. # and ## indicate a significant enhancement in the bioenergetic parameter, compared to the control group (no AP39) (*P* < 0.05 and *P* < 0.01, resp.); *∗* indicates a significant reduction in the bioenergetic parameter, compared to the control group (*P* < 0.05).

Similar to the responses previously noted for authentic H_2_S [25, 33, 34], exposure of WT neurons to AP39 induced a significant increase in the basal OCR at 100 nM but induced a decrease in the basal OCR at 250 nM (Figure 2(a)). Additionally, AP39 caused a dose-dependent increase in the FCCP-stimulated OCR at 25 and 100 nM but caused a decrease in the FCCP-stimulated OCR at 250 nM (Figure 2(b)). The FCCP-induced increase in the OCR represents the “maximal respiration.” Thus, AP39 acts as a supplemental bioenergetic stimulator at low concentrations. The pattern of the effects of AP39 is consistent with the previously established bell-shaped pharmacological properties of H_2_S [19, 35]. These results demonstrated that this inhibitory effect is only conspicuous at higher concentrations (Figure 2(b)).

### 3.3. AP39 Exerted Cytoprotective Effects on APP/PS1 Neurons

**Figure 3:**
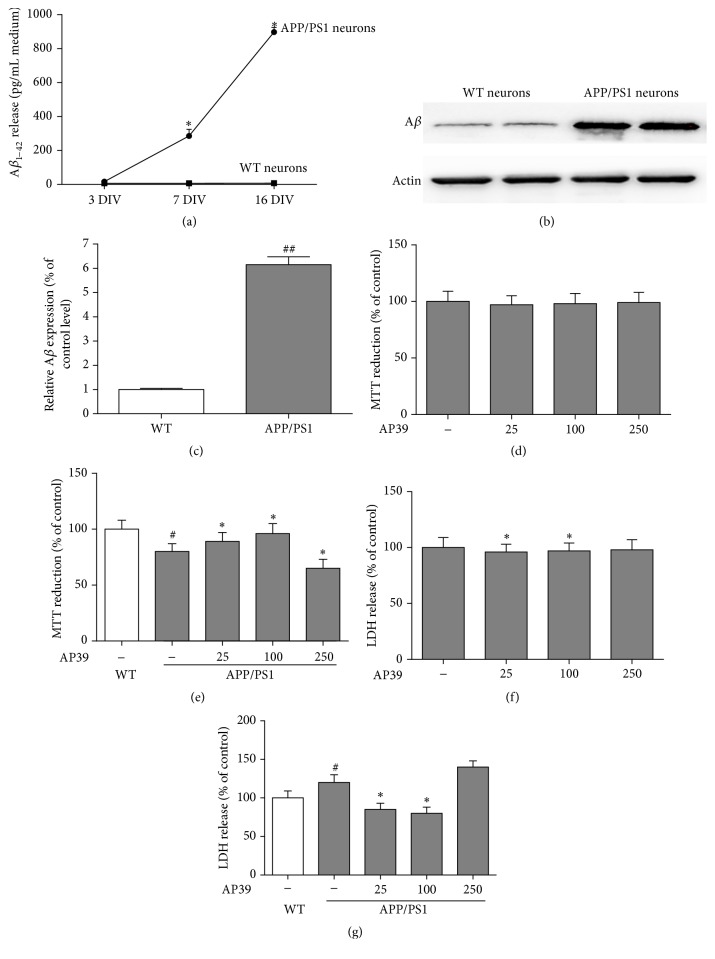
Cytoprotective effects of AP39 in APP/PS1 neurons. (a) A time course shows that increasing levels of A*β*_42_ are released into the culture media of neurons from transgenic mice but not their WT littermates. (b) A*β* was overexpressed in neurons from APP/PS1 mice compared to A*β* expression in neurons from their WT littermates. (c) Representative of A*β* blots is shown with quantification. (d) The effects of AP39 (25–250 nM) treatment for 24 h on cell viability in WT neurons. AP39 alone did not affect MTT conversion. (e) The effects of AP39 (25–250 nM) on MTT conversion in APP/PS1 neurons. There was a decrease in MTT conversion in the APP/PS1 neurons compared to the WT neurons; these effects were attenuated by AP39. (f) After treatment of WT neurons with AP39 for 24 h, AP39 alone did not affect LDH release in the cellular medium. (g) The effects of AP39 on LDH release from APP/PS1 neurons. ^*∗*^ *P* < 0.05, versus the APP/PS1 group; ^#^ *P* < 0.05, the APP/PS1 group versus the WT group.

We cultured cortical neurons from the brains of embryonic APP/PS1 transgenic mice as an experimental model to study the effects of AP39. We detected the levels and time course of the release of A*β*_42_ from these neurons into the culture media by ELISA.  Figure 3(a) shows that at 3 DIV a small amount of A*β*_42_ was released into the media of neurons cultured from APP/PS1 mice, but significant differences were observed between neurons cultured from APP/PS1 mice and their WT littermates (16.1 ± 6.9 and 5.9 ± 2.7 pg/mL, resp.). By 7 DIV, the level of A*β*_42_ produced in the cultures from the APP/PS1 mice was significantly higher than that produced in the cultures from the WT mice (285.7 ± 38.6 versus 6.2 ± 0.7 pg/mL, *P* < 0.01), and this level was further increased at 16 DIV (898.3 ± 27.3 versus 6.8 ± 0.5 pg/mL, *P* < 0.01). The total protein from WT and APP/PS1 neurons was extracted separately and analyzed by Western blot. We observed that A*β* was overexpressed in the APP/PS1 primary neurons compared to the WT neurons (Figures 3(b) and 3(c)).

Exposure of WT neurons to AP39 for 24 h had no effect on cell viability, but treating the APP/PS1 neurons with AP39 (25,100 nM) for 24 h resulted in increase in cell viability (Figures 3(d) and 3(e)). To confirm this result, we measured the release of LDH in the supernatant of the primary neurons. AP39 had no effect on basal LDH release in WT neurons, but there was a significant increase in the amount of LDH in the APP/PS1 neuron culture medium. This result indicated a loss of cell membrane integrity and an increase in cell necrosis. In contrast, after treatment with AP39, particularly at 100 nM, the amount of LDH in the APP/PS1 neuron culture medium was decreased (Figures 3(f) and 3(g)).

### 3.4. AP39 Attenuated the Loss of Cellular Bioenergetics and Protected Mitochondrial Function in APP/PS1 Neurons

**Figure 4:**
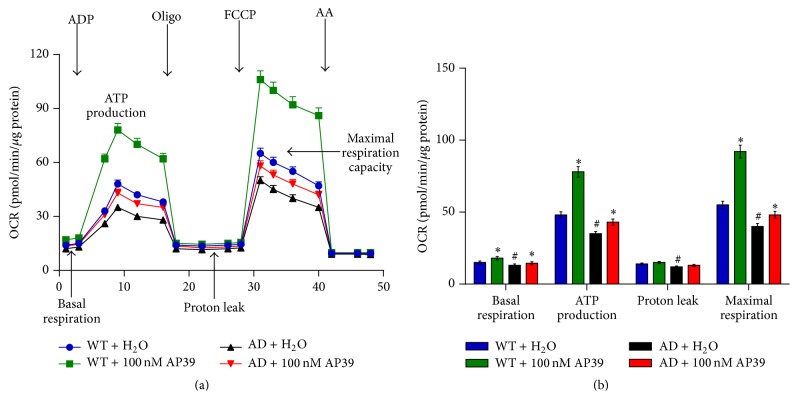
Protective effects of AP39 on the cellular bioenergetics of APP/PS1 neurons. APP/PS1 neurons were incubated in AP39 (100 nM) for 24 h, and bioenergetic parameters were measured using the Extracellular Flux Analyzer. (a) Representative tracings are shown. (b) The calculated bioenergetic parameters are shown. *∗* indicates a significant enhancement of the bioenergetic parameter compared to the control (H_2_O) (*P* < 0.05); # indicates a significant reduction in the bioenergetic parameter compared to the control (WT + H_2_O) (*P* < 0.05).

To investigate the effects of AP39 on mitochondrial dysfunction, we measured cellular bioenergetics using the XF24 Extracellular Flux Analyzer. Cellular bioenergetic parameters were significantly decreased in the mitochondria of APP/PS1 neurons compared with those of WT neurons. Importantly, AP39 (100 nM) significantly increased the basal respiratory rate and the OCR-linked maximal respiratory capacity of the APP/PS1 neurons (Figure 4). These data indicated that AP39 may attenuate the loss of cellular bioenergetics in APP/PS1 neurons.

**Figure 5:**
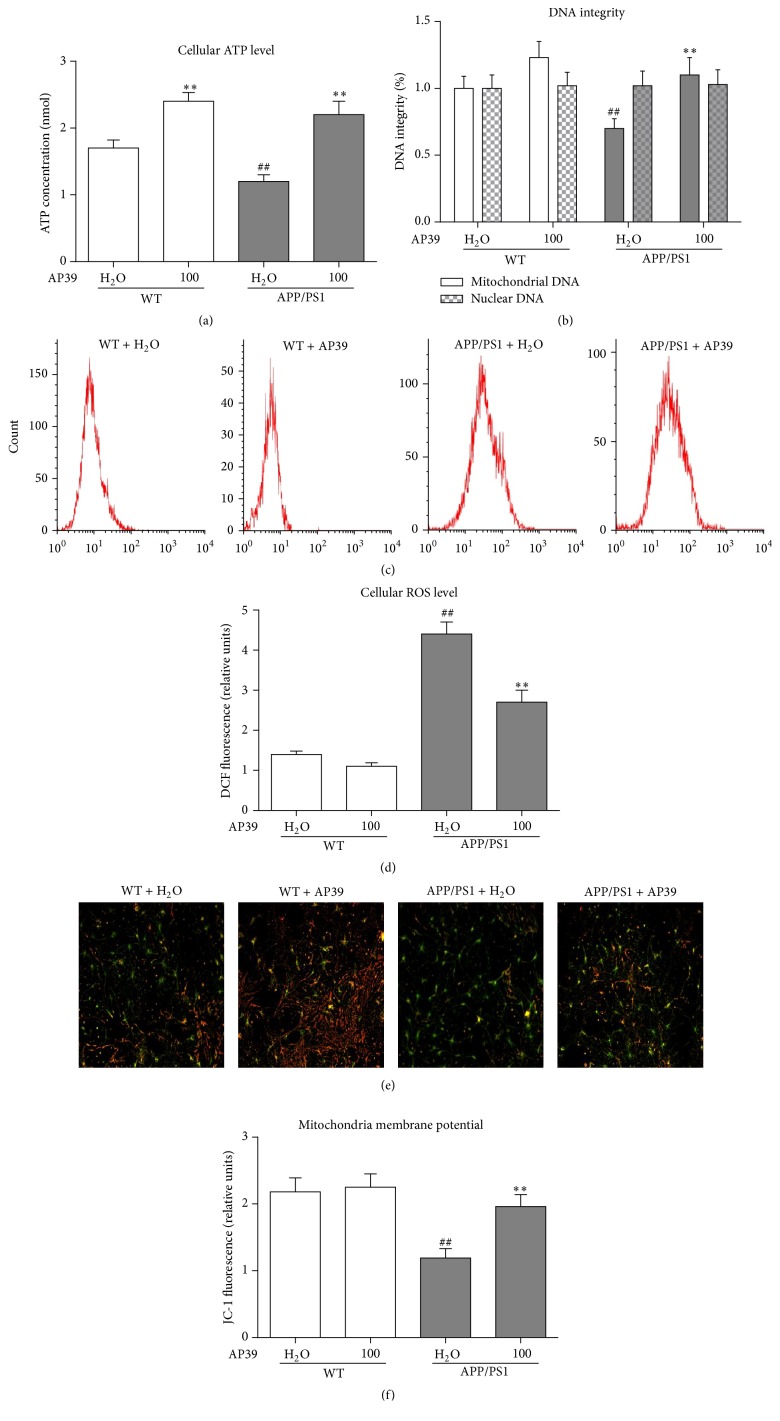
AP39 protected mitochondrial function in APP/PS1 neurons. (a) AP39 increased the cellular ATP levels. (b) AP39 protected mtDNA integrity. (c) ROS levels were detected by flow cytometry. (d) AP39 reduced ROS levels. ^##^ *P* < 0.01, compared with the WT group; ^*∗∗*^ *P* < 0.01, compared with the control group (H_2_O).

ATP production, mitochondrial DNA (mtDNA) integrity, and ROS production were measured to evaluate mitochondrial function. AP39 significantly increased the ATP production in WT and APP/PS1 neurons (Figure 5(a)). Next, mtDNA and nuclear genomic DNA integrity was assessed via PCR of long DNA fragments. We found that mtDNA but not nuclear DNA integrity was clearly reduced in APP/PS1 neurons, compared to WT neurons. However, AP39 significantly protected against mtDNA damage in APP/PS1 neurons by partially restoring mtDNA integrity (Figure 5(b)).

Intracellular ROS levels are a basic indicator of oxidative stress. A fluorescence assay using a DCFH-DA probe was performed to quantify the generation of ROS. AP39 (100 nM) effectively decreased ROS levels in APP/PS1 neurons (Figures 5(c) and 5(d)). In conclusion, these results suggested that AP39 protects against mitochondrial dysfunction in APP/PS1 neurons.

### 3.5. AP39 Shifted the Mitochondrial Dynamics toward Fission in APP/PS1 Neurons

**Figure 6:**
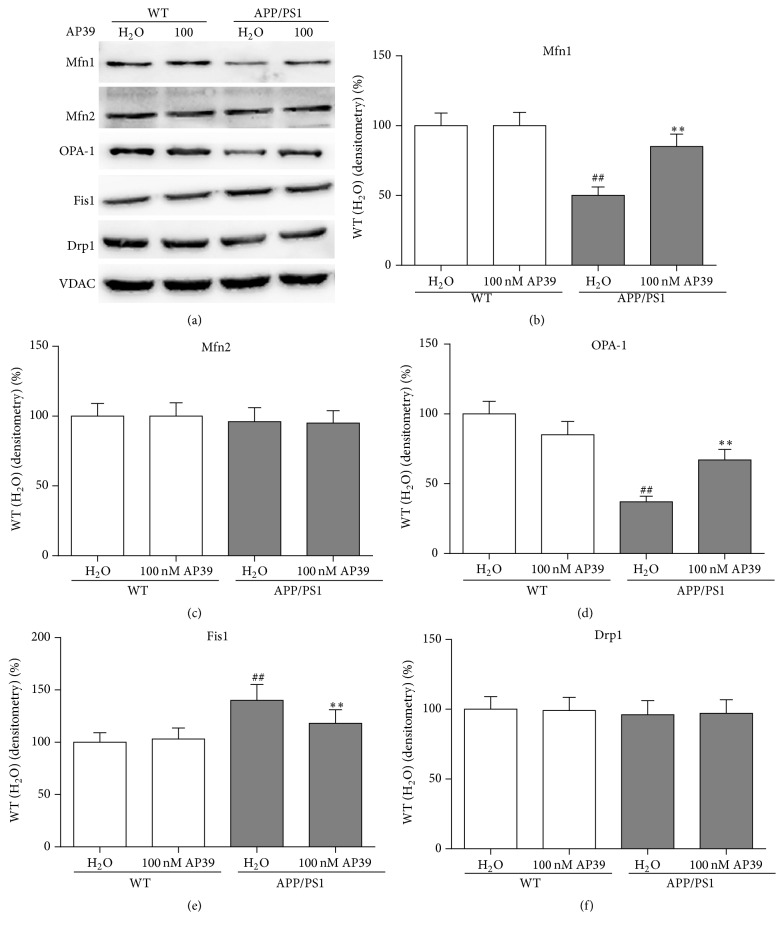
AP39 reduced the expression of proteins involved in mitochondrial fusion but increased the expression of a mitochondrial fission protein. (a) Protein samples were probed for Drp1, Fis1, Mfn1, Mfn2, OPA-1, and VDAC expression. (b–f) Representative blots are shown with quantification. ^##^ *P* < 0.01 AD mice receiving water compared to WT mice receiving water; ^*∗∗*^ *P* < 0.01 mice receiving AP39 compared with mice receiving water.

The changes in the levels of proteins involved in mitochondrial dynamics (Drp1, Fis1, Mfn1, Mfn2, and OPA1) were determined via Western blot analysis (Figure 6). The levels of these mitochondrial dynamics-related proteins were shifted toward fission in APP/PS1 neurons. The levels of the mitochondrial fusion proteins Mfn1 and OPA1 were significantly reduced, and the level of the mitochondrial fission protein Fis1 was markedly increased in APP/PS1 neurons compared with WT neurons. The protein levels of Mfn2 and Drp1 were not significantly different between APP/PS1 and WT neurons. OPA1, Mfn1, and Mfn2 catalyze mitochondrial fusion. AP39 increased the levels of OPA1 and Mfn1 but not Mfn2. Moreover, AP39 decreased the levels of Fis1 but not Drp1. These findings suggested that AP39 may shift mitochondrial dynamics in APP/PS1 neurons from fission towards fusion.

### 3.6. AP39 Increased the Generation of H_2_S in WT and APP/PS1 Mice

**Figure 7:**
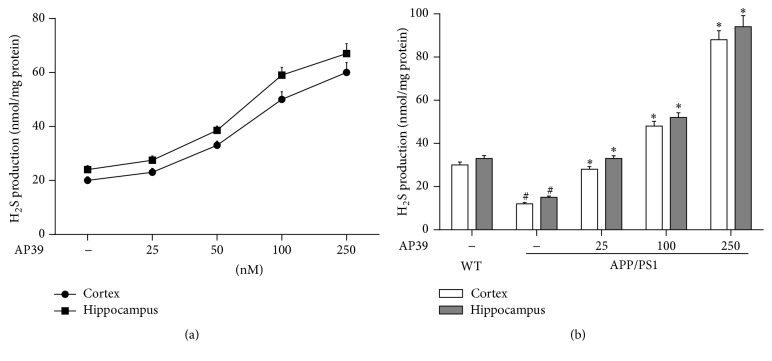
AP39 generates H_2_S in WT mice and AP39 increased H_2_S levels in APP/PS1 mice. (a) The contribution of AP39 to H_2_S production in the cortex and hippocampus of WT mice. (b) AP39 increased H_2_S levels in APP/PS1 mice. ^##^ *P* < 0.01, compared with the WT group; ^*∗∗*^ *P* < 0.01, compared with the control group (H_2_O).

AP39 (25–250 nM) induced a dose-dependent increase in H_2_S generation in the cortex and hippocampus of WT and APP/PS1 mice (Figures 7(a) and 7(b)). We also observed that H_2_S levels in the cortex were lower than those of the hippocampus. However, the level of H_2_S was significantly decreased in APP/PS1 mice compared to WT mice. In conclusion, the data indicate that AP39 contributes to the synthesis of H_2_S in WT and APP/PS1 mice.

### 3.7. AP39 Reversed the Memory Deficits of APP/PS1 Transgenic Mice

The APP/PS1 transgenic mice developed a pronounced spatial learning and memory deficit and produced A*β* plaques by 12 months of age [22]. To test the ability of AP39 to ameliorate the spatial learning deficits of the AD model mice, we treated 12-month-old AD model mice and WT controls with 100 nM/kgAP39. Then, behavioral testing was initiated 6 weeks after the initiation of AP39 therapy using the Morris water maze and the NORT.

**Figure 8:**
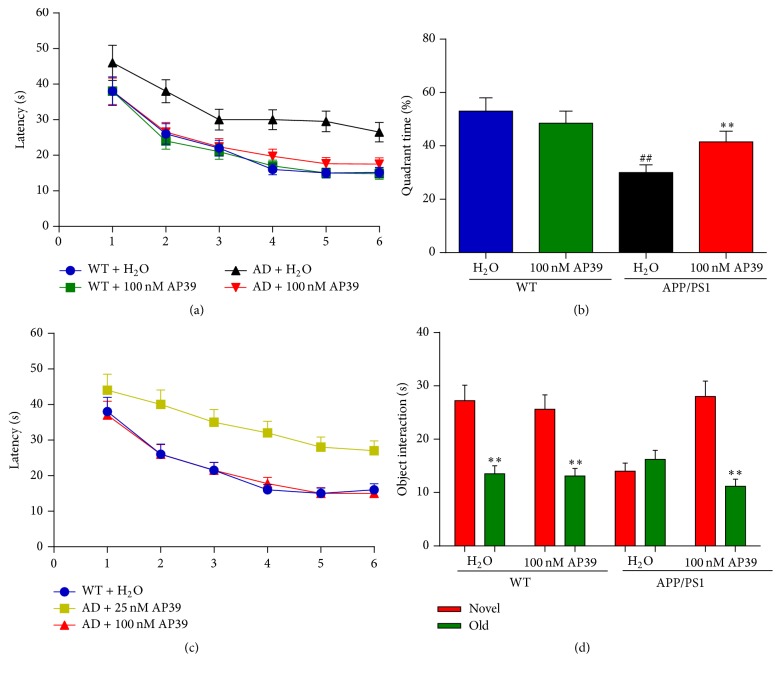
AP39 ameliorated the learning and memory deficits of APP/PS1 mice. WT or AD mice were treated with water or AP39 (100 nM/kg) for 6 weeks, followed by assessment using the Morris water maze and novel object recognition task tests. (a) Spatial learning and memory in AD mice are scored as the latency to locate a hidden platform. (b) After 24 h, a 60-s probe trial was performed. (c) Spatial learning was tested as the latency to locate a hidden platform for the 25 nM/kg AP39-treated mice and scored. (d) Effects of AP39 on the memory performance of 12-month WT or AD mice treated with water or AP39 were tested in the NORT test.

In the training trials, treatment with AP39 for 6 weeks eliminated the preexisting deficits of the transgenic mice, as demonstrated by a reduced latency to locate the hidden platform compared to the latency of the WT control with AP39 (Figure 8(a)). The WT mice with or without AP39 treatment preferred the target quadrant in the probe trial. In contrast, the AD model mice treated with H_2_O spent much less time in the target quadrant, near the chance level of 15 s. The AD model mice treated with AP39 showed the same strong preference for the target quadrant as the WT mice. Thus, after 6 weeks, AP39 treatment reversed the spatial learning and memory deficits of the aged AD model mice (Figure 8(b)). This benefit was concentration-dependent, as 25 nM/kg AP39 treatment did not improve the water maze performance of the AD model mice (Figure 8(c)). Next, the same mice with 100 nM/kg AP39 or H_2_O treatment were subjected to the NORT test (Figure 8(d)). The NORT test does not put stress on the animal and does not require spatial orientation. It has been used to measure deficits in learning and memory in various AD mouse models [36, 37]. Importantly, AD mice treated with water performed poorly and presented an impairment compared to the WT mice, indicated by a significant reduction in the percentage of time exploring the novel object; this behavior was consistent with an impairment in memory function for familiarization on the previous day. In contrast, the AP39-treated AD mice showed significantly improved performance levels compared to the water-treated AD mice level. Thus, treatment of aged APP/PS1 mice with 100 nM/kg AP39 for 6 weeks reversed the age-dependent memory impairment. All of these results indicated that appropriate doses of AP39 improved the learning and working memory ability of AD model mice.

### 3.8. AP39 Inhibited the Brain Atrophy of APP/PS1 Transgenic Mice

**Figure 9:**
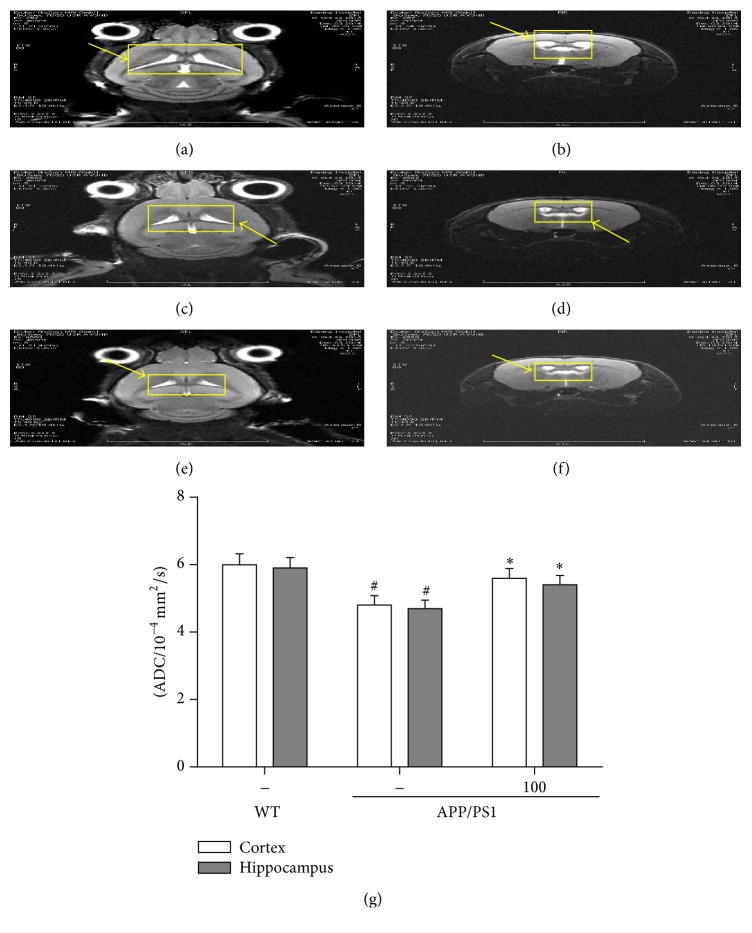
AP39 alleviated the brain atrophy of APP/PS1 mice as observed by brain magnetic resonance imaging (MRI). Brain MRI data were collected at different coronal and axial sections in 12-month-old mice receiving different treatments. Notes: panels (a), (c), and (e) show coronal sections, and panels (b), (d), and (f) show axial sections. The highlighted white regions indicate cerebrospinal fluid (CSF) in the ventricle, and the hippocampus is adjacent to the ventricle. Representative images from brain MRI depict slices of the T2-weighted morphologic images of 12-month-old WT mice treated with water (a)-(b) or AD mice treated with water (c)-(d) or AP39 (e)-(f). (g) ADC value determination of the mouse brains.

In addition to behavioral assessments, mice were randomly selected for intravital scanning to noninvasively study brain structure using MRI data. We studied the coronal and axial sections of the mouse brains (Figures 9(a)–9(f)). As expected, there were no apparent structural abnormalities in the WT mouse brains. For example, the sizes of the ventricle were symmetric between the two brain hemispheres, and the edges of the ventricle were clear and sharp (Figures 9(a) and 9(b)). However, we observed visible atrophy in the brains of the 12-month-old AD model mice treated with water (Figures 9(c) and 9(d)). Their ventricles were asymmetric, with a much larger size on the right than the left side, and had unclear edges. The MRI imaging data revealed that AP39 might alleviate brain atrophy and ventricle asymmetry in AD model mice (Figures 9(e) and 9(f)).

The apparent diffusion coefficient (ADC) value in parietal cortex and hippocampus of each group of mice was compared with diffusion weighted imaging (DWI) sequences.  Figure 9(g) showed the ADC value in parietal cortex and hippocampus of APP/PS1 mice significantly declined compared to those in WT mice, whereas AP39 significantly increased the ADC values in parietal cortex and hippocampus of APP/PS1 mice (*P* < 0.05).

### 3.9. AP39 Reduced the Levels of A*β* and A*β* Deposition in APP/PS1 Transgenic Mice

**Figure 10:**
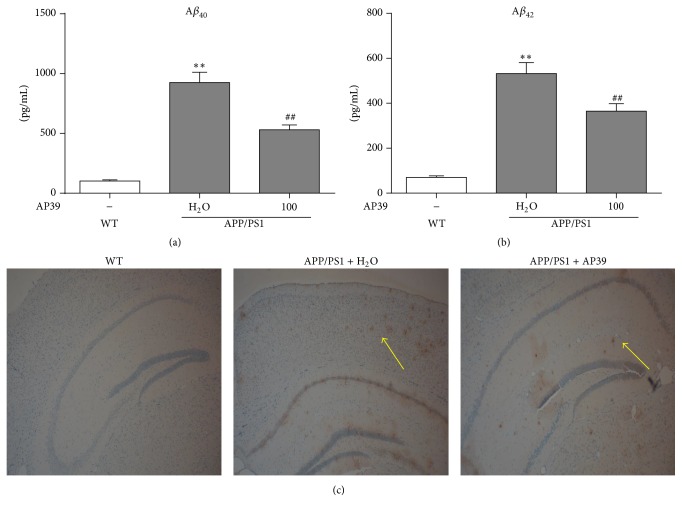
AP39 reduced A*β* production and A*β* deposition in APP/PS1 mice. (a)-(b) After intraperitoneal injection of AP39 for 6 weeks, A*β*_40_ and A*β*_42_ levels in the mouse brains were decreased from 925 pg/mL and 532 pg/mL to 531 pg/mL and 365 pg/mL, respectively. (c) A*β* plaques were detected in the cortex and the hippocampus of the WT and APP/PS1 mouse brains based on 6E10 immunostaining. The statistical results show the area of the A*β* plaques. Original magnification: –40x; scale bars = 100 *μ*m.

After different treatments for 6 weeks, A*β* levels and plaque development were examined in vivo. The A*β*_40_ and A*β*_42_ levels in the 12-month-old AD transgenic model mouse brains were 925 and 532 pg/mL, respectively. Importantly, treatment with 100 nM/kg AP39 decreased the A*β*_40_ and A*β*_42_ levels in AD mice compared to water treatment (Figures 10(a) and 10(b)). As expected, amyloid plaques were not observed in WT mice. However, intracellular amyloid in the hippocampal and cortical tissues of the AD model mice treated with water was remarkably increased compared with the WT control mice. This observation is consistent with those from a previous study [31]. Importantly, treatment with AP39 significantly reduced both the number and the size of intracellular amyloid plaques in the cortical and hippocampal tissues compared to treatment with water in the AD mice (Figure 10(c)). The amyloid plaque burden in the brains of AD model mice treated with AP39 was decreased compared with the brains of AD mice treated with water (Figure 10(c)). These results demonstrated the ability of AP39 to inhibit A*β* plaque deposition in the brains of AD mice.

## 4. Discussion

Our findings demonstrate that AP39, a newly synthesized mitochondrially targeted H_2_S donor, maintains cellular bioenergetics and exerts mitochondrial protective effects in AD neurons and mice. First, AP39 exerted concentration-dependent modulatory effects on cell viability and cellular bioenergetic function. At 25 or 100 nM, AP39 enhanced cell viability and bioenergetics, but the highest AP39 concentration (250 nM) reduced these parameters in APP/PS1 neurons. In addition, treatment with an appropriately selected concentration of AP39 (100 nM) increased ATP levels, protected mtDNA integrity, decreased intracellular ROS levels, and regulated mitochondrial dynamics. Moreover, AP39 significantly inhibited brain atrophy and ameliorated the memory deficits and A*β* deposition in APP/PS1 mice.

H_2_S, which has recently been determined to be the third most abundant gasotransmitter, plays a variety of physiological and pathological roles in the central nervous system and other systems [38, 39]. An increasing amount of evidence suggests that H_2_S is a potential therapeutic drug for AD. Recently, it was reported that H_2_S-modulating agents such as S-ally-l-cysteine (SAC) and Tabiano spa waters (enriched in H_2_S) protected against impairments in learning and memory in AD transgenic mice by modulating inflammation and apoptosis [40–42]. In addition, our group reported that NaHS can promote the nonamyloidogenic processing of APP and improve spatial learning and memory acquisition in APP/PS1 mice [43]. However, due to technical limitations for H_2_S detection, at present its biological roles and metabolites in vivo have remained elusive, resulting in considerable controversy [44]. Moreover, the use of sulfide salts such as NaHS is limited, although the solutions to these problems have recently become apparent [36]. Importantly, recent studies indicated that AP39 acts as an endogenous H_2_S donor and exerts antioxidative and cytoprotective effects on bEnd.3 cells [19]. AP39 decreased blood pressure, heart rate, and pulse wave velocity in rats [37]. Based on these limitations and progress, we synthesized AP39 to evaluate its protective effects on mitochondrial function in APP/PS1 mice.

We observed that AP39 increased H_2_S levels in neurons and mitochondria. Given that AP39 has the TPP+ group, which has been used to selectively target various molecules [45–47], this resulted in AP39 successfully and selectively delivering H_2_S to mitochondria. This result is consistent with previous literature [19].

Previous research demonstrated that H_2_S can act as an electron donor and as a potential inorganic source of energy in mammalian cells, resulting in an increase in cellular oxygen utilization and ATP production [48, 49]. Subsequently, studies reported opposing (stimulatory at lower concentrations; inhibitory at higher concentrations) effects of H_2_S on cellular bioenergetics in HT-29 Glc(−/+) cells and some immune cells [50, 51]. However, owing to the various measurement methods used in experiment, the exact concentrations of H_2_S reported to induce stimulation versus inhibition were different. Recently, Módis et al. reported that 3-MST-derived H_2_S functions as an endogenous bioenergetic factor that donates electrons to SQR: the subsequent mitochondrial electron transport is coupled to aerobic ATP generation. In Hepa1c1c7, low concentrations of H_2_S (0.1–1 *μ*M) elicited a significant increase in mitochondrial electron transport and cellular bioenergetics and higher concentrations of H_2_S (3–30 *μ*M) were inhibitory [18]. In our experiments, AP39 (25–100 nM) induced the stimulation of cellular bioenergetics and AP39 (250 nM) induced marked inhibition in WT neurons. Moreover, APP/PS1 neurons exhibited higher “baseline” rates of oxygen consumption, electron transport, and cellular bioenergetic status than WT neurons. However, it is noted that AP39 (100 nM) increased the basal respiratory rate and exerted a protective effect on the cellular bioenergetics of the APP/PS1 neurons. These results are consistent with a previous study showing a stimulatory role for H_2_S on bioenergetics but are opposed to the inhibitory effects of H_2_S on mitochondrial electron transport at complex IV [52–54], which occurs at supraphysiological concentrations. Interestingly, the concentrations of AP39 (nM) that resulted in either stimulation or inhibition were lower than those previously reported using H_2_S. We considered that AP39 accumulated in mitochondria, in which the AP39 concentration is likely substantially higher than the “nominal” concentration that was applied to the culture medium, resulting in this discrepancy. Overall, these data indicated that the stimulatory and inhibitory effects of H_2_S occur in a narrow concentration range (in a specific concentration range to exert positive bioenergetic effects; exceeding this range produces inhibitory effects). However, the exact molecular mechanism underlying the protective effect of AP39 remains to be investigated in further experiments.

It has been reported that transgenic mouse strains harboring a single (APP) or double (APP and PSEN1) genetic mutation generate amyloid plaques [55]. In our experiments, A*β*_42_ release was significantly decreased in neurons cultured from APP/PS1 mice compared to neurons from WT mice, and this result may be attributed to these two (APP and PSEN1) gene mutations. Previous reports have commonly used A*β*PP cultured primary neurons to evaluate A*β*PP processing and A*β* secretion [56, 57] and have shown that A*β* accumulates intracellularly in A*β*PP cultured neurons [58]. Our data are consistent with the reports of these previous studies.

We first evaluated the effects of AP39 on APP/PS1 neurons. In APP/PS1 neurons, cell viability was decreased and LDH release was improved compared to WT neurons. The results may be attributable to the fact that media from the A*β*PP primary neuronal cultures contained toxic components that led to neuritic degeneration [59]. However, AP39 improved the situation in a dose-dependent manner. These findings demonstrate the beneficial effects of AP39.

Recently, mtDNA damage and oxidative stress were found to play important roles in the pathogenesis of AD [60]. A*β* can decrease mitochondrial energy production, increase ROS levels within the mitochondria, and injure sensitive mtDNA [61]. In our study, treatment of APP/PS1 neurons with AP39 was shown to increase neuronal ATP production and reduce ROS levels. Interestingly, the APP/PS1 neurons showed significant damage to their mitochondrial DNA, and this damage may be due to A*β* plaque deposition in these neurons. More importantly, we found that AP39 clearly increased mitochondrial, but not nuclear, DNA integrity. Our results suggest that AP39 may partially contribute to the enhancement of mitochondrial biogenesis, the repair of oxidative damage, and the attenuation of mitochondrial dysfunction in AD. However, we can not exclude the possibility that H_2_S may also affect the integrity or activity of various mitochondrial DNA repair proteins in APP/PS1 neurons. This potential relationship remains to be explored in further experiments. Recently, accumulating evidence has suggested that there is an imbalance in mitochondrial fission and fusion in AD progression [62, 63]. Our study showed that the levels of the fission proteins Mfn1 and OPA1 were significantly decreased but that the levels of the fusion protein Fis1 were markedly increased in APP/PS1 neurons. These results suggested that mitochondrial fission and fusion were altered in APP/PS1 neurons. However, no apparent changes in the levels of the mitochondrial fusion protein Mfn2 or the mitochondrial fission protein Drp1 were observed in the APP/PS1 neurons. This result was consistent with the observation of mitochondria with increased fission and decreased fusion based on the related mRNA and protein levels in A*β*PP primary neurons [9]. Importantly, AP39 treatment reversed these changes in APP/PS1 neurons. The molecular basis for the extensive changes in mitochondrial fusion and fission observed in neurons in response to the pathogenesis of mitochondrial-related neurodegenerative diseases has not been described [64]. Interestingly, our data are not consistent with the findings of all previous reports. These inconsistencies may be due to the use of a different experimental model and different environments in vivo and in vitro. Importantly, AP39 treatment reversed these changes in APP/PS1 neurons. Whether the three genes exhibiting altered expression mediate the effects of AP39 on AD is currently under investigation.

To test whether AP39 protects AD model mice against impaired cognitive function, we examined both spatial memory and object recognition memory in APP/PS1 mice. After six weeks of AP39 treatment, the age-dependent memory deficit in the APP/PS1 mice was fully rescued on both memory tests: AP39 ameliorated their spatial learning and memory impairments, as shown by faster movement to the target quadrant of the pool. The reduced swimming time near the periphery of the pool indicated that AP39 can help to relieve anxiety, a common symptom of AD, in mice. In the present study, AP39 (100 nM/kg) was a more effective therapeutic concentration than AP39 (25 nM/kg). The dose-dependence of the observed memory improvement implies that an appropriate dose of AP39 is required for its beneficial effects on learning and memory. It remains possible that higher doses of AP39 are deleterious to memory function.

Due to its advantages, such as noninvasiveness and the ability to perform repeated in vivo measurements, MRI has been widely used in AD research [65] and in the auxiliary clinical diagnosis of AD [66]. It was used to directly visualize the inner structure of the intravital cerebrum and to measure the in vivo changes in brain volume [67], amyloid burden [68], and white matter. The hippocampus is a brain area critical for learning and memory and is especially vulnerable to damage in the early stages of AD cases [69]. In our study, the changes in the ventricle, including the deepening and widening of the sulci and the gyri, were clearly observed by MRI. In the water-treated AD mice, we observed asymmetrical ventricles, which indicated the atrophy of regions surrounding the ventricle. However, in the AP39-treated AD mice, we observed that the ventricles were symmetrical and exhibited clear edges. Because the hippocampus is adjacent to the ventricle, we hypothesized that AP39 may partially prevent hippocampal atrophy. However, we must further investigate this hypothesis. Our data suggest that MRI is a useful tool to observe the dynamic neuropathology changes in AD mouse brains and those changes in aged AD mice observed by MRI correlated with their cognitive dysfunction in the current study.

Interestingly, recent research has indicated that the mitochondrial translocation of H_2_S-producing enzymes produces an endogenous protective response to various insults [70].

In our study, AP39 served as a pharmacological tool or potential therapeutic agent that “mimics” this endogenous protective mechanism. However, some limitations of this study should be noted. First, we have not precisely determined the exact cellular metabolism of AP39. It will be necessary to conduct additional studies to establish the dynamics of the cellular metabolism of AP39 in the future. Second, the bell-shaped dose-response properties of AP39 are similar to those observed for authentic H_2_S or other H_2_S donors, and we must admit that there may be additional pharmacological mechanisms that may be conducive to the detected decrease in cellular bioenergetics at higher concentrations of AP39. The above limitations must be studied in future experiments.

## 5. Conclusions

Taken together, the results of the current study revealed that the main target of protection by AP39 was mitochondria based on the APP/PS1 model. We showed that AP39 exerts concentration-dependent modulatory effects on cellular bioenergetics in APP/PS1 neurons. In addition, AP39 increased ATP levels, decreased oxidative damage to mtDNA, and reduced ROS levels in APP/PS1 neurons. Moreover, AP39 regulated the balance between mitochondrial fission and fusion. All of these events contribute to an attenuation of mitochondrial dysfunction in APP/PS1 neurons. Based on these data, AP39 exerts multiple protective effects in the APP/PS1 model, including the amelioration of memory deficits, the prevention of brain atrophy, and the decrease of A*β* deposition. Therefore, AP39 represents a good candidate disease-modifying therapy for AD. Despite recent advancements in our understanding of the role of AP39 in mitochondrial bioenergetics and function, whether AP39 is actually beneficial for AD patients remains to be determined and warrants further study.
